# Optimizing the photon ratio of red, green, and blue LEDs for lettuce seedlings: a mixture design approach

**DOI:** 10.1186/s13007-023-01098-8

**Published:** 2023-11-05

**Authors:** Eunjeong Lim, Jong-Ok Kim, Myung-Min Oh

**Affiliations:** 1 Division of Animal, Horticultural and Food Sciences, Chungbuk National University, Cheongju, 28644 Republic of Korea; 2 Brain Korea 21 Center for Bio-Health Industry, Chungbuk National University, Cheongju, 28644 Republic of Korea; 3 School of Electrical Engineering, Korea University, Seoul, 02841 Republic of Korea

**Keywords:** Optimization, Lettuce seedlings, LED, Light quality, Mixture design, Response surface

## Abstract

**Background:**

Light control technology has been developed and studied for decades in controlled environment agriculture (CEA) for successful crop production. The effects of the light spectrum on plant growth can vary because plants have spectral specific responses, and mixed light elicits interactive combination effects. Response surface methodology (RSM) can be utilized with the design of experiments to optimize a response influenced by multiple inputs with limited data. In this study, we aimed to identify the optimal photon ratio in combination of red (R), green (G), and blue (B) light-emitting diodes (LEDs) for growing lettuce seedlings using RSM and a seedling-indicating parameter by performing a similarity analysis of response surfaces that elucidated the response tendency of different factors, such as light quality.

**Results:**

The highest shoot fresh weight was obtained from the R treatment (red LED 100%) at the end of the seedling stage. However, the RGB_141_ (photon ratio of R:G:B = 1:4:1) treatment during the seedling stage resulted in the highest shoot fresh weight at the final harvest. The value of the leaf area multiplied by the leaf chlorophyll concentration (SPAD) was selected as the seedling-indicating parameter. The optimal RGB photon ratio that maximized this parameter was R:G:B = 30.6:44.0:25.4, and this ratio was verified by conducting identical cultivation experiments. During the first 6 days after transplanting, SPAD gradually increased in R-treated seedlings, while the optimal treatment maintained the value at a higher constant level, which supported our result of shoot fresh weight at harvest.

**Conclusions:**

Thus, we confirmed that the mixture design method allowed us to optimize the combined RGB photon ratios for the seedling stage in order to maximize the growth index of mature lettuce plants and to select an appropriate seedling-indicating parameter that represents the final harvest results to benefit crop production in CEA.

**Supplementary Information:**

The online version contains supplementary material available at 10.1186/s13007-023-01098-8.

## Background

Controlled Environmental Agriculture (CEA) can lead to high and uniform crop production throughout the year by tailoring environmental conditions to plant requirements in insulated and airtight places. The use of only artificial light in CEA is advantageous for maintaining set conditions without interruption from fluctuating solar light [1]. However, unlike free and ubiquitous solar light, the production of artificial light consumes energy, which can account for 70–80% of total electricity costs [2]. Therefore, optimizing artificial lighting conditions for crop production and equipment operation is vital to improve the efficiency of light use. Light, an important resource in crop production systems, can induce various physiological and morphological changes in plants. Environmental factors, such as light intensity or carbon dioxide concentration, which are strongly correlated with plant growth, are usually optimized by a regression model with a response (plant growth) to a single factor (e.g., light intensity, carbon dioxide concentration) [3, 4]. However, the light quality is difficult to optimize due to the independent and combined effects of its various wavelength ranges, such as far-red, red, green, blue, and ultraviolet light, resulting in complex effects on plant growth and development [5]. In the range of photosynthetically active radiation (PAR, 400–700 nm), red light promotes responses such as stem elongation and leaf expansion, whereas blue light regulates de-etiolation, chloroplast movement, and stomatal opening, such that red and blue light elicit opposite effects on leaf thickness and area [6]. In contrast, green light is not efficiently absorbed by chlorophylls and acts antagonistically to blue light by inducing the conversion of cryptochrome forms. Furthermore, the influence of green light could be interpreted differently at the leaf or canopy level [7, 8].

In general, light quality experiments are reported as comparisons between phenotypic or genetic differences under different light treatments. However, research on optimizing the light quality condition tends to involve too many treatments and produce results that require complex interpretation due to the characteristics of light quality mentioned above. In this sense, Response Surface Methodology (RSM), one of the optimization methods, can be used with continuous values obtained from surface regression based on a few treatments. In addition, the Design of Experiments (DOE) technique is used to design experimental components, such as treatment, data collection, and statistical methods, to obtain the maximum amount of information with a minimum number of replications [9]. When the response is affected not only by the combination ratio of components (factors) but also by their amounts, the mixture design method, a type of DOE technique, can be used to identify an optimal condition with a smaller number of treatments [10]. Since its development in the 1950s, RSM has been used in various fields, such as analytical chemistry, for optimization [11]. In plant science, RSM was used to optimize the isolation and transfection conditions of maize endosperm protoplast, which was useful for characterizing unidentified related genes [12]. Bredda et al. [13] reported optimization research using a mixture design methodology for microalgae production under different light spectrum conditions.

Seedling production in vertical farms and greenhouses is usually carried out in a separate location with a higher plant density and under specific environmental conditions, such as a lower electrical conductivity of the nutrient solution and higher relative humidity, because of the growth responses of seedlings, which are different from those of mature plants [14]. The definition of good seedling quality includes the ability to adapt to the main cultivation area after transplanting, which is related to seedling growth characteristics such as hypocotyl diameter, leaf color, and root zone development [15]. An important factor affecting seedling quality is light quality due to its role in photosynthesis and photomorphogenesis [16]. Several studies have reported correlations between seedling growth characteristics induced by light spectral control and mature plant growth [17, 18]. Although the effect of light quality on plant morphology at both seedling and after transplanting stages has been well established, there is limited information on which and how light quality-induced growth characteristics affect after transplanting.

**Fig. 1:**
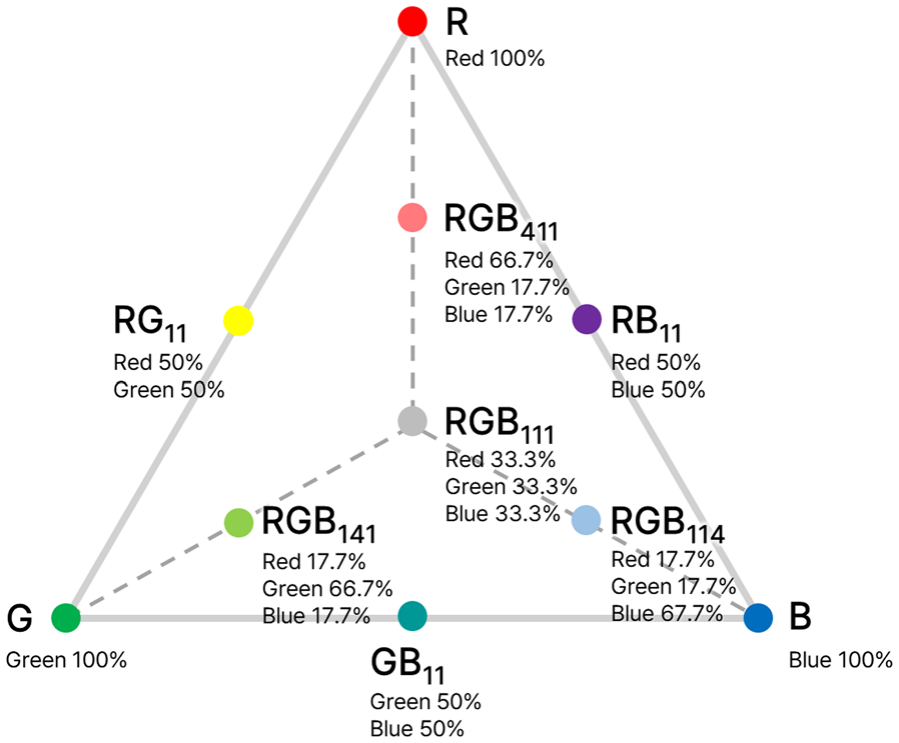
RGB light treatments designed by simplex axial mixture design. The light intensity of all treatments was the same, with different combination ratios of red, green and blue LED lights. Monochromatic light as red (R), green (G) and blue (B) lights, dichromatic light as R:G = 1:1 (RG_11_ ), R:B = 1:1 (RB_11_ ) and G:B = 1:1 (GB_11_ ) and trichromatic light as R:G:B = 4:1:1 (RGB_411_ ), R:G:B = 1:4:1 (RGB_141_ ), R:G:B = 1:1:4 (RGB_114_ ) and R:G:B = 1:1:1 (RGB_111_ )

**Fig. 2:**
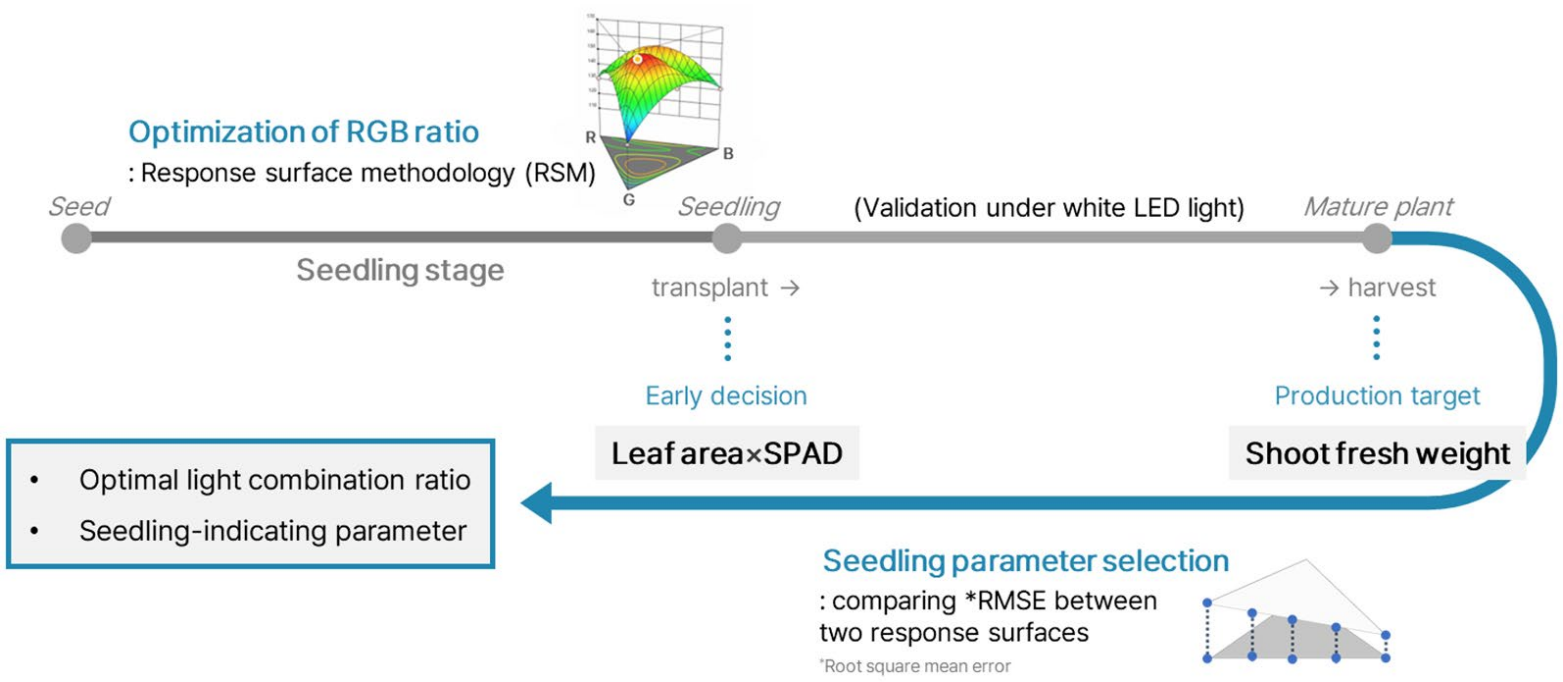
A workflow of this study. The light treatments were applied during the seedling stage to optimize the RGB photon ratio using response surface methodology. The optimal light for the seedling stage was depended on the shoot fresh weight of the mature plant at harvest, which was set as the production target. The selection of seedling parameters was conducted by comparing the root square mean error value between two response surfaces, shoot fresh weight at harvest and one of the seedling growth parameters. The value of multiplying the leaf area and SPAD of seedlings was selected and can be used for early decisions regarding seedling production

The aim of this study was to determine an optimal photon ratio of red (R), green (G), and blue (B) LED light for the seedling phase of lettuce production using RSM. The experiments consisted of experiment 1 (exp. 1) as a baseline experiment with ten different light spectra (Fig. 1), which was set up with a simplex axial design of the mixture design to find the optimal R:G:B photon ratio and experiment 2 (exp. 2), which was carried out to verify the optimal photon ratio under identical cultivation conditions. In this process, the optimal conditions for seedling production were determined by studying the production yield of the mature lettuce plant under extended cultivation and white LED conditions. By analyzing the growth characteristics of seedlings and mature plants, our findings were obtained and suggest a new growth parameter indicating seedling quality. Finally, we introduced a methodology to determine the optimal light spectrum using LEDs through a mixture design method (Fig. 2).

## Results

### Seedling growth characteristics and optimization of the RGB photon ratio

**Fig. 3:**
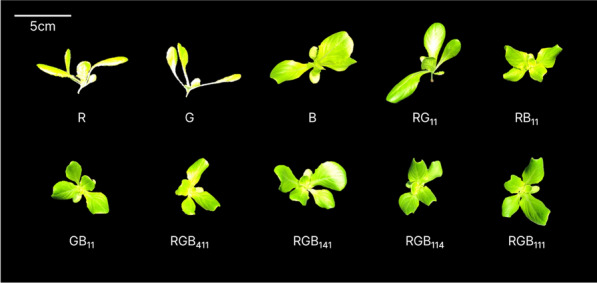
Top-view images of lettuce seedlings at 2 weeks of 10 different light treatments based on mixture design. Scale bar: 5 cm

**Fig. 4:**
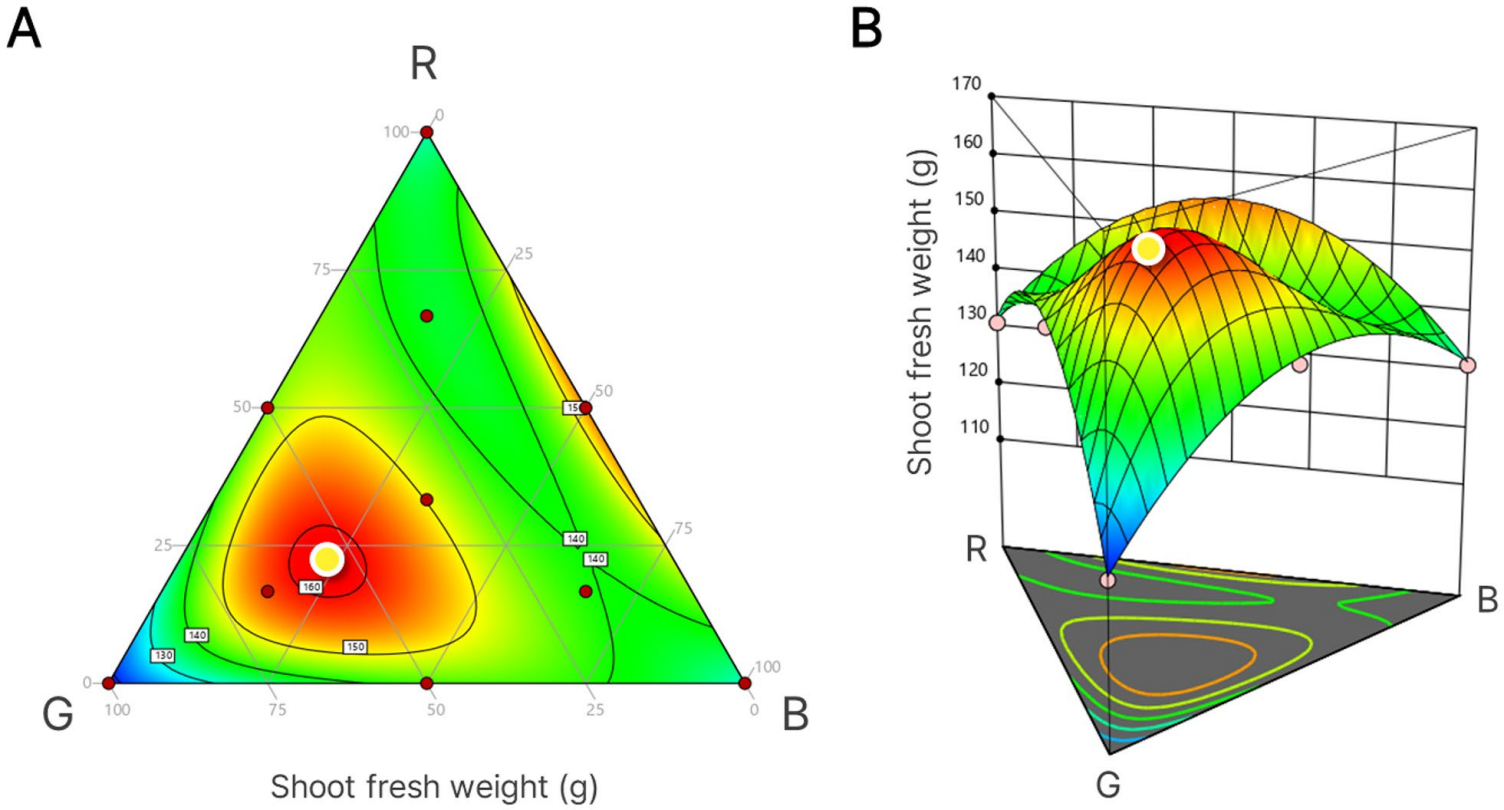
Response surface methodology (RSM) results based on the shoot fresh weight of mature plants treated with white LEDs for 4 weeks of cultivation after 2 weeks of 10 light treatments; a contour plot (**A**) and three-dimensional image (**B**). Yellow dots in **A** and **B** show optimal points on response surfaces for shoot fresh weight

After light treatment during the seedling stage, the shoot fresh weight of lettuce seedlings under monochromatic (mono) R showed the highest value among all treatments. RGB_411_, which has the highest proportion of red light after R, resulted in a shoot fresh weight that was 1.32 times lower than that of R, but the shoot fresh weight of RGB_141_-treated plants was 1.56 times higher than that of G-treated plants. No significant differences were detected in the case of blue light (RGB_114_ and B) (Table 1). The seedling leaf area showed a trend similar to shoot fresh weight. However, the group with the highest seedling leaf area was RG_11_, RGB_411_, RGB_141_, and RGB_111_, as well as R. Combinations without blue light (R, G, and RG_11_) tended to have lower SPAD values, which represent chlorophyll content per unit leaf area. However, only R had no significant differences compared to other treatments with B. Specific leaf area (SLA) was lower in R than in the other treatments, except for RG_11_ and RB_11_. RGB_141_ had the highest SLA value among all treatments. Seedlings under the R and G treatments had elongated hypocotyls that could not easily stand vertically, as well as elongated leaves (Fig. 3). Seedlings treated with R and G had lower projected leaf areas than those treated with B, RG_11_, RGB_141_, and RGB_111_ (Table 1). Mature lettuce plants treated with RGB_141_ at the seedling stage showed the highest mean value of shoot fresh weight among all treatments. For the RSM results shown in Fig. 4, the yellow point indicates the highest value on the response surface (maximum z value). The corresponding RGB photon ratio at this point was R:G:B = 21.7:55.1:23.2.

**Table 1:** Growth parameters of lettuce seedlings after 10 light treatments during the 2-week seedling stage

	Shoot fresh weight (g)	Root dry weight (mg)	Leaf area (cm^2^)	Specific leaf area (cm^2^ g^−1^)	Projected leaf area (cm^2^)	SPAD
R	0.60a	38.7	16.5a	27.7d	12.0d	26.7ab
G	0.33e	31.2	10.2d	31.6abc	10.0d	20.5d
B	0.41cd	55.6	13.4bc	32.7abc	25.8a	29.4ab
RG_11_	0.49b	32.9	15.2ab	30.8bcd	18.1b	22.9cd
RB_11_	0.38cde	42.1	11.3cd	29.9cd	10.3d	30.5a
GB_11_	0.37de	47.1	11.5cd	30.9abcd	13.1cd	27.9ab
RGB_411_	0.50bc	38.1	14.7ab	32.7abc	12.6d	29.7a
RGB_141_	0.50b	36.9	17.3a	34.4a	16.7bc	25.6bc
RGB_114_	0.43bcd	32.7	13.6bc	32.0abc	14.0bcd	28.9ab
RGB_111_	0.50b	25.9	17.5a	33.6ab	17.5b	29.1ab

Different letters indicate statistically significant differences among light treatments at *p* < 0.001 based on ANOVA and Duncan’s multiple range test (n = 5)

### Selection of seedling indicating parameter

**Fig. 5:**
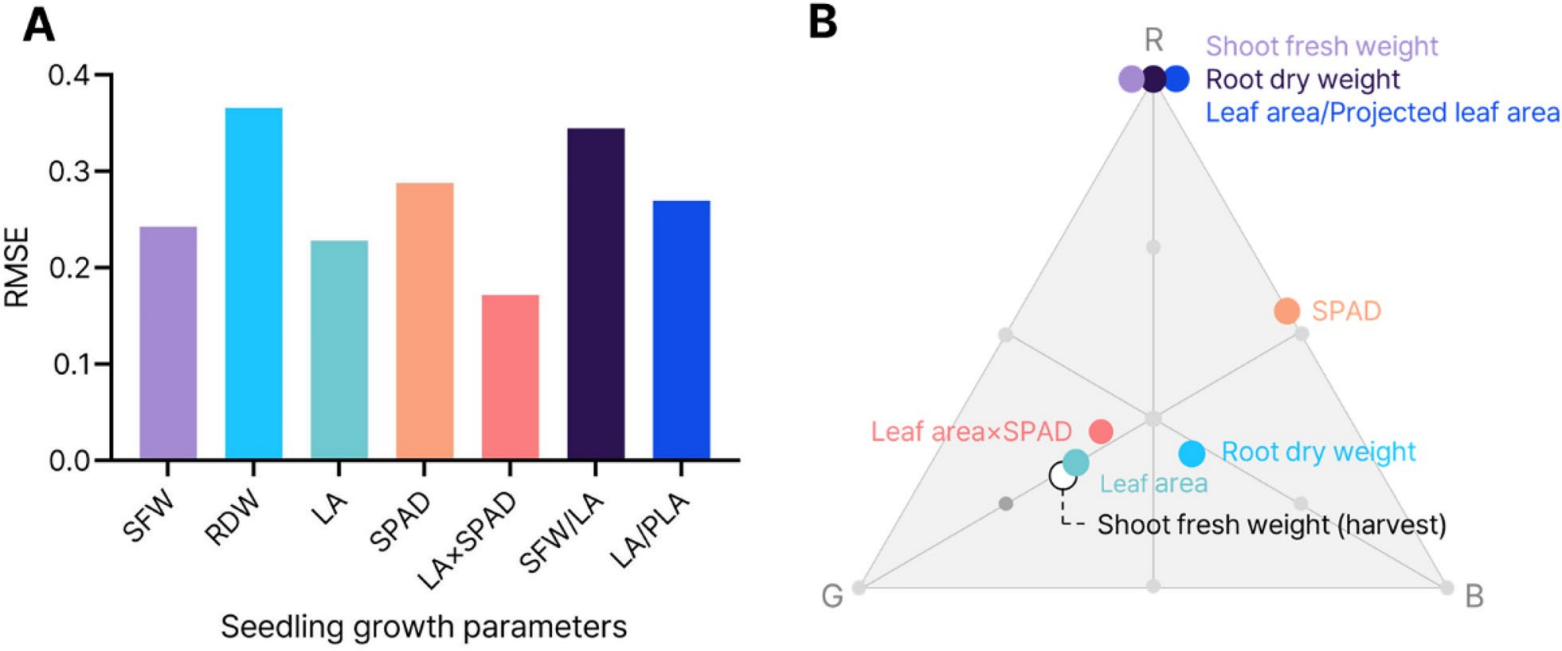
Root mean square error (RMSE) value between response surfaces based on the shoot fresh weight of mature plant at harvest and seedling growth parameters: shoot fresh weight (SFW), root dry weight (RDW), leaf area (LA), SPAD, LA×SPAD, SFW/LA and LA/projected leaf area (PLA) (**A**). All response value data were normalized for comparison. Their position of optimal combination ratio based on each parameter is shown on a triangle that has red (R), green (G) and blue (B) lights as three vertexes (**B**)

Each response surface based on the growth parameters of RGB light-treated seedlings was used for similarity analysis with a response surface from the shoot fresh weight of mature lettuce plants at harvest. Among the seedling growth parameters of single and multiple measured properties, LA×SPAD, which indicates leaf area and SPAD multiplied by the same ratio, showed the lowest RMSE (root mean square error) value (Fig. 5A). Figure 5B shows the optimal RGB photon ratios for the growth parameters. The optimal RGB photon ratio was R:G:B = 100.0:0:0 for seedling shoot fresh weight and R:G:B = 30.6:44.0:25.4 for LA×SPAD. Despite its optimal point being closer to that of the shoot fresh weight at harvest, the leaf area parameter had a higher RMSE value than LA×SPAD.

### Verification of optimal RGB lights

**Fig. 6:**
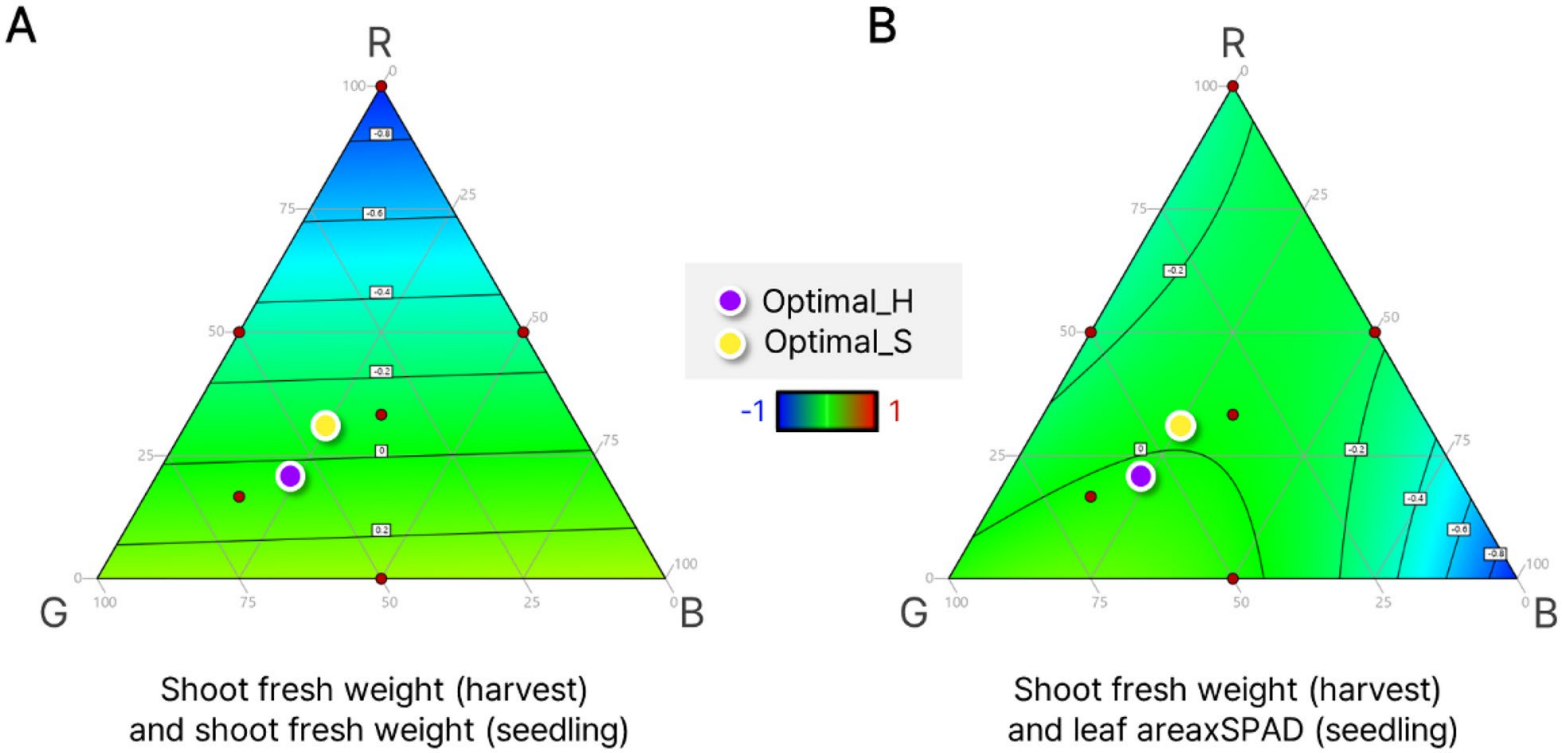
Plots of differences in shoot fresh weight at harvest and shoot fresh weight at seedling (**A**) and leaf area×SPAD at seedling (**B**). All three response values were normalized from 0 to 1, and their differences were fitted by response surface methodology. The scale of color represents red and blue for higher contrast and green for lower contrast. The purple and yellow dots on the plots indicate the optimal photon ratio from shoot fresh weight at harvest (Optimal_H) and leaf area×SPAD at seedling (Optimal_S), respectively

The optimal RGB photon ratio based on LA×SPAD at the seedling stage (Optimal_S) and shoot fresh weight at harvest (Optimal_H) were confirmed in exp. 2. Figure 6 shows the differences between shoot fresh weight (A) and LA×SPAD (B) at seedling stages and shoot fresh weight at harvest, the cultivation target. Three parameters were each normalized from 0 to 1 in advance. The red points in the plots indicate the treatments. Figure 6B, based on LA×SPAD, shows a green color, which means a 0 difference value in all areas except for the B-axis area, which was not used for light treatment in exp. 2. However, there was a significant difference in shoot fresh weight between seedlings and harvest in the R-axis area (Fig. 6A).

### Comparison of red and optimal light treatments after transplanting

**Fig. 7:**
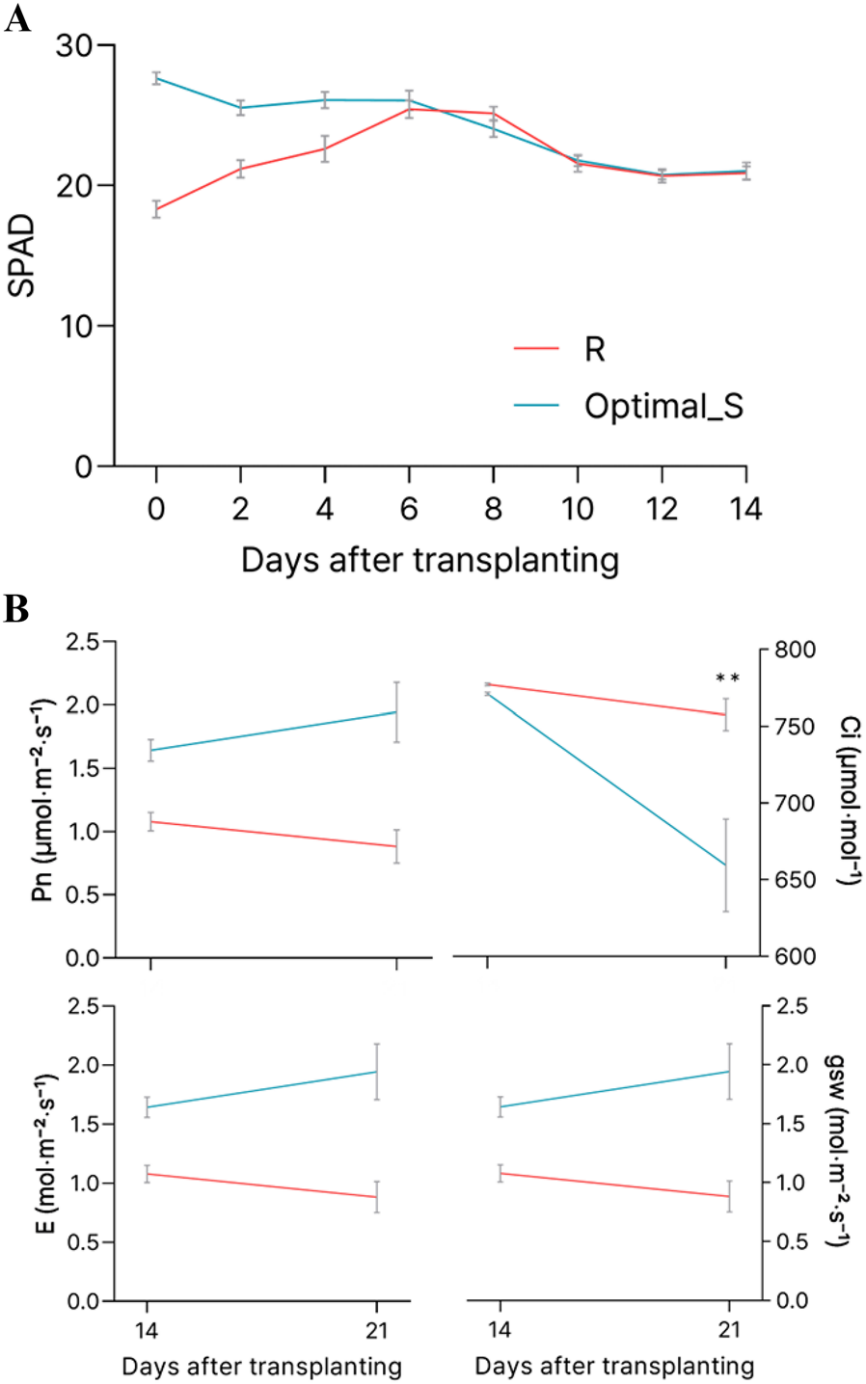
Variation in SPAD value over 2 weeks after transplanting seedlings grown under mono red (R) and an optimal photon ratio based on leaf area×SPAD parameter at seedling (Optimal_S) light treatments to white LED in experiment 2 (**A**). The change in SPAD value after transplanting was measured every 2 days for 2 weeks for the verification experiment (n = 4). Photosynthetic rate (Pn), transpiration rate (E), intercellular CO_2_ concentration (Ci), and stomatal conductance (gsw) of lettuce plants grown under mono R and Optimal_S light during the seedling stage at 2 and 3 weeks after transplanting (n = 4) (**B**). ** indicates significant differences at *p* < 0.01 based on Student’s t-test

During cultivation in exp. 2, seedlings treated with R showed increasing SPAD values right after being transplanted and placed under white LED conditions until 8 days after transplanting (DAT) (Fig. 7). In contrast, the SPAD values of the Optimal_S treatment were high compared to those of R treatment until 6 DAT. A decreasing trend in SPAD values was observed for both treatments from 6 or 8 DAT. Eventually, the SPAD values of both treatments were almost identical and maintained at approximately 20 from 10 to 14 DAT. In terms of photosynthetic characteristics, the intercellular CO_2_ concentration (Ci) at 3 weeks after transplanting (WAT) was significantly lower for Optimal_S than for R. The photosynthetic rate (Pn), transpiration rate (E), and stomatal conductance (gsw) of Optimal_S showed a higher tendency than those of R.

## Discussion

The response values for each growth-related parameter on the RGB triangle (Fig. 1) can appear on a response surface as fitted by regression models. Therefore, the RGB combination ratio for the highest value on a response surface is suggested as optimal, which has been difficult to determine with existing simple comparison experiments [11]. In our study, after 2 weeks of ten different light treatments, it was found that seedling shoot fresh weight found to be highest under R by simple comparison among ten treatments (Table 1), consistent with the RSM result. Because red light is effectively used for photosynthesis, it can accumulate high biomass [19]. The development of hypocotyl elongation in the absence of blue light showed a result consistent with the generally known role of blue light (Fig. 3) [20]. Prolonged hypocotyl elongation may compromise the ability of the stem to support leaf weight, resulting in morphological abnormalities, abnormal shape, and reduced light-capturing ability. However, leaf expansion induced under red and green light (or in the absent of blue light) may be related to the beneficial effect of increased carbon assimilation through enhanced light capture [21]. Therefore, there are many considerations when selecting light conditions for proper plant growth and development.

The three-dimensional shape of the response surface shows the effects of the light components (R, G, and B LED lights) on the response values; therefore, the response of the growth-related parameter responds to these LED lights was expressed as a response surface equation. In other words, the similarity of the response to light quality may be highly related to the similarity of the shape of the response surfaces. ‘LA×SPAD’ at the seedling stage had the lowest RMSE value of the target shoot fresh weight at harvest, and its optimal RGB photon ratio was R:G:B = 30.6:44:25.4, which was between that of RGB_141_ and RGB_111_. Its availability as an optimal RGB photon ratio for the seedling stage was estimated to be not significantly different from Optimal_H at both the seedling stage and harvest in exp. 2 (data not shown). These results suggest that a parameter in the middle of cultivation with a similar response surface to the shoot fresh weight at harvest may be critical for achieving maximum production yield. This methodology can also be applied to target parameters other than shoot fresh weight and even more than one growth index as multiple parameters by overlaying the responses. By assigning different weights to each parameter, growers can utilize it according to their cultivation goals. For example, when we considered both shoot fresh weight and leaf area as a multiple growth index, the result showed that the optimal ratio was changed by setting weights for the parameters (Additional file 1: Fig. S2).

The optimal RGB photon ratio (R:G:B = 30.6:44.0:25.4) selected for the seedling stage consisted of all three light spectra (R, G, and B), with a relatively high proportion of green light (44%) in our study. Although green light alone had a negative effect on seedling growth, a positive effect was observed when red and blue light were present, even at low levels. The RGB_141_, which contains 16.7% each of red and blue light, had a different effect on shoot fresh weight and leaf area than mono G. The leaf area of G-treated plants was the lowest, while that of RGB_141_ plants was the highest among the ten light treatments (Table 1). In Arabidopsis photoreceptor mutants, green light induces shade avoidance-like phenotypes, including leaf expansion in the presence of background red and blue light [22]. In addition, the shade avoidance response is induced by the inversion of cryptochrome 1, which is activated by blue light and converted to inactivated oxidized flavin by green light [23]. Red and blue light, which are strongly absorbed by chlorophyll pigments, have important functions in photosynthesis and related photomorphogenic reactions [24]. They are mainly absorbed by the upper chloroplasts of mesophyll cells due to their high absorption rate [25]. While the high absorption and quantum yield of red light resulted in the highest shoot fresh weight at the seedling stage, weakly absorbed and highly transmitted green light has been shown to reach spongy parenchyma tissues below the upper chloroplasts, enhancing the absorption rate of RGB light at the whole leaf level [7]. In this study, as 200 µmol m^−2^ s^−1^ light was applied at both the seedling and cultivation stages, the amount of red and blue light may be sufficient to saturate the upper chloroplasts of the seedling leaves. In addition, the PPFD conditions were set based on empty cultivation beds and identically for both seedling and after transplanting stages. Therefore, it is expected that the photon flux reaching the leaf surface of the plants would have differed as the plants grew over the cultivation period. The quantitative ratios of the blue light range of 400–500 nm wavelength, the green light range of 500–600 nm, and the red light range of 600–700 nm can be influenced by the optical characteristics of the light source, such as its peak wavelength. These characteristics can cause variations in the absorption spectra of plant pigments, including chlorophyll. The peak wavelengths of the RGB LEDs used in this research differed from those used in previous reports yet elicited similar plant responses. This suggests that plants may have a broader range of sensitivity to RGB lighting than previously thought: (R) 660 nm, 665 nm [20], 630 nm [22], (G) 525 nm [22], (B) 467 nm, 468 nm [20], 470 nm [22]. However, such considerations may be necessary for more sophisticated analysis and quantitative approaches to light treatment.

The mean shoot fresh weight of seedlings was greater under R compared to Optimal_S and Optimal_H in exp. 2. However, during transplanting to harvest, after 4 weeks of uniform cultivation under white LEDs, the optimal light treatments surpassed R in terms of the same parameter (shoot fresh weight at harvest). This inconsistency highlights the significance of matching the parameter to the plant growth stages. Using our response surface similarity results, we selected LA×SPAD at the seedling stage as the most appropriate parameter for predicting our target trait: shoot fresh weight at harvest. A larger leaf area is advantageous for light interception by the plant in terms of photosynthesis. Light quality and intensity both influence leaf area by regulating leaf expansion level or leaf number, as observed in photoinhibition and shade avoidance. Green light is known to have a role in leaf expansion [22]. As an increase in leaf area can be induced by morphological responses and an accumulation of photosynthetic assimilates, mono G treatment resulted in a smaller leaf area than RGB_141_ (Table 1) due to the absence of red and blue lights required for photosynthesis. The group with the highest leaf areas (R, RG_11_, RGB_411_, RGB_141_, and RGB_111_) included the R treatment, resulting in the highest shoot fresh weight. It also caused seedlings to develop elongated hypocotyls and petioles (Fig. 3). Since plants capture light as three-dimensional structures, seedlings under R treatment could be at a disadvantage compared to those exhibiting normal structural development, which is crucial for growth. Thus, there may be a discrepancy between a single leaf area parameter and the final harvest shoot growth results. A number of studies have reported the potential effects of given treatment conditions during the seedling stage on mature plant growth and yield [17, 26–28]. Although they received the identical light treatment after transplanting, the light conditions during the seedling stage partially affected final growth [25, 27]. In particular, the improvement of seedlings’ light capturing ability had a positive effect on harvest point. Additionally, SPAD values, a non-destructive measure of chlorophyll content, is another parameter for seedling growth. Previous studies have reported a strong correlation (R^2^ > 0.9) between SPAD and chlorophyll content [29, 30]. When plants were treated with blue light, the SPAD value increased compared with that under red light [31]. Moreover, as the proportion of blue light increased, the chlorophyll content also increased in cucumber plants [32]. The absence of blue light may cause leaf expansion but also hinder chlorophyll accumulation. In total, the seedlings with a large leaf area and high chlorophyll content exhibited that effective light interception and photosynthetic apparatus development were advantageous for ultimate yield of production. A combination photon ratio of R, G, and B lights was established through a mixture design method to attain these qualities.

How seedlings acclimate after transplanting led to differences in growth properties at the seedling and harvest stages. In other words, various growth characteristics of seedlings were found to be associated with their acclimation performance immediately after transplanting. When comparing the R treatment, which resulted in the highest shoot fresh weight at the seedling stage, and the Optimal_S treatment, which produced the most significant yield at harvest, contrasting changes in SPAD patterns were observed during the first 6 days. In the first 6 days after transplanting, Optimal_S maintained high SPAD values, unlike R (Fig. 6). Because SPAD was measured per unit area of a leaf, the differences in total chlorophyll content between R and Optimal_S-treated plants would be more significant, given the growth and leaf expansion during that period. According to Yang’s (2014) research on the temporal dynamics of SPAD readings over the plant lifespan; SPAD values generally increase during the growth stage to maintain the functionality and decrease during senescence [33]. Thus, LA×SPAD reflects the seedlings’ growth state and potential to acclimate to new environmental conditions and thrive after being transplanted. Assuming that there is horticulturally beneficial potential in CEA to dynamically adjust environmental conditions suitable for the plant growth status throughout the growth period, an additional goal for future cultivation technology could be to explore cultivation conditions that facilitate rapid acclimatization or adaptability to dynamically changing environmental conditions.

The analysis of the connections between various environmental factors in the current static environmental control method will yield much more complex results with the application of dynamic control. As an instance, artificial light sources provide light energy to plants as well as affect air temperature within a cultivation space in CEA. Although air conditioning was used in this study to reduce temperature differences between treatment conditions, in optimizing light quality the energy consumed by light sources and used to control the heat generated by the light sources should also be considered in the future. The growth trend observed under various light quality conditions in this study was supported by numerous previous studies on crop growth responses to light conditions, given that the optimized RGB photon ratio determined through RSM is reliable. Furthermore, representing the plant growth parameter for the combination of light quality in three wavelength ranges as a response surface could perform a more complex analysis involving similarities at different time points. Although our findings share limitations with previous studies, caused by various environmental factors and plant varieties, we anticipate that gathering more data in diverse conditions and utilizing the strengths of this methodology will enable more detailed analyses of interactions spanning various environmental conditions and genetic factors.

## Conclusion

The photon ratio from red, green, and blue LEDs was optimized with the mixture design of RSM. Ten light spectra based on a simplex axial design were used during the seedling stage. These seedlings were then transplanted and exposed to general white LEDs for phenotypic analysis until harvest. Moreover, the response surfaces of single or multiple growth-related parameters at the seedling stage were compared with those of the shoot fresh weight at harvest. The most identical parameter with the lowest RMSE value at 250 points of surfaces was deemed the indicating parameter at the seedling stage. The LA×SPAD value was found to be the ideal parameter for seedlings, while the optimal RGB photon ratio was R:G:B = 30.6:44.0:25.4 (Optimal_S). There were no significant differences observed regarding optimal RGB photon ratios based on the shoot fresh weight at harvest (Optimal_H). Validation experiments indicated that both optimal light treatments exhibited the highest seedling LA×SPAD and shoot fresh weight at harvest. Different RGB response patterns at each time point signified the necessity of an indicator parameter in the middle of cultivation to inform dynamic lighting strategies. Thus, we determined the optimal ratios of RGB photons and indicating parameters during the seedling stage of lettuce plants. Taken together, our findings demonstrate the practicality and effectiveness of mixture design for the advancement of crop cultivation.

## Experimental materials and methods

### Light conditions

During the 2 weeks of the seedling stage, ten light treatments with a simplex axial design were radiated using red, green, and blue LEDs that had peak wavelengths at 665, 518, and 451 nm, respectively; monochromatic lights: R, G, and B; dichromatic lights: R:G = 1:1 (RG_11_), R:B = 1:1 (RB_11_), and G:B = 1:1 (GB_11_); and trichromatic lights: R:G:B = 4:1:1 (RGB_411_), R:G:B = 1:4:1 (RGB_141_), R:G:B = 1:1:4 (RGB_114_), and R:G:B = 1:1:1 (RGB_111_) (Additional file 1: Fig. S1). The spectral distribution of the red LED did not include the far-red range (700–750 nm). These ten light treatments were used for the first experiments (exp. 1) and two optimal treatments (Optimal_S and Optimal_H) from exp. 1 were added in later experiments (exp. 2) without B and RGB_114_. The ratios of red, green, and blue light were calculated and set with sums of 600–700 nm, 500–600 nm, and 400–500 nm, respectively, using a JAZ-EL 200 spectroradiometer (Ocean Optics, Dunedin, FL, USA). The total light intensity and period of all treatments were identical, with 200 µmol m^−2^ s^−1^ photosynthetic photon flux density (PPFD) and 16 h (light)/8 h (dark). PPFD was adjusted using a quantum meter (LI-1400, LI-COR, Lincoln, NE, USA), and the measurements of all light treatments were based on the 9 points of empty cultivation bed without plants.

### Plant materials and growth conditions

Experiments were conducted with butterhead lettuce ‘Fairly’ (Enza Zaden, Enkhuizen, The Netherlands) on a hydroponic module. Seeds were sown on polyurethane sponges (2.5 cm × 2.5 cm) thoroughly soaked in distilled water, and 72-cell trays, including the sponges, were covered with transparent lids to achieve over 90% relative humidity (RH) for 3 days. Each tray was placed in the middle of cultivation spots (80 cm × 68 cm × 10 cm), separated for ten different light treatments. The following environmental conditions were maintained for 2 weeks of seedling growth: 23 °C, 80% RH, and a CO_2_ concentration of 500 µmol mol^−1^. The temperature, humidity (Vaisala HMP60 sensor, Vaisala, Vantaa, Finland), and CO_2_ concentration (KCP-HP100, Korea digital, Seoul, Republic of Korea) in the cultivation room were monitored every minute and regulated to set values by a data logger (CR1000; Campbell Scientific, Inc., Logan, UT, USA) connected to sensors. Three fans were installed in each cultivation spot to produce 0.74 ± 0.09 (mean ± standard error) m s^−1^ of airflow (Testo 405i hot-wire anemometer, Titisee-Neustadt, Germany). Two liters of lettuce nutrient solution (Sonneveld and Straver, 1994) at pH 5.8 with an electrical conductivity (EC) of 0.8 dS m^−1^ were provided to each tray and replenished every 3 days.

After 2 weeks of growth, eight seedlings per treatment were transplanted to nutrient film technique system cultivation beds. Seedlings were arranged in a completely randomized design. The planting distance between individuals was 12 cm. Lettuce nutrient solution (pH 5.8, EC 1.2 dS m^−1^) applied at the seedling stage was subirrigated and calibrated using a pH·EC sensor (WTW Multi 3430 IDS; WTW GmbH, Weilheim, Germany) every 3 days. For the 4 weeks after transplanting, the cultivation conditions were 20 °C, 70% RH, a CO_2_ concentration of 800 µmol mol^−1^, and an airflow of 0.81 ± 0.12 m s^−1^. White LEDs (Bissol LED, Seoul, Republic of Korea) were used as a light source with the same light intensity (200 µmol m^−2^ s^−1^) and period (16 h).

### Growth parameters

Seedlings treated with ten different light spectra for 2 weeks were investigated for shoot fresh and dry weights, root dry weight, leaf area, projected leaf area, and SPAD value. Shoot fresh and dry weights and root dry weight were measured with an electronic scale (Si-234, Denver Instrument, NY, USA). To measure dry weight, shoots and roots of lettuce plants were dried in an air oven at 70 °C for over 3 days. Leaf area was measured using a leaf area meter (LI-3100, LI-COR). Specific leaf area was calculated by dividing the total leaf area by shoot fresh weight. The projected leaf area was obtained from a top-view image of plants by segmented pixels of the G channel in MATLAB (R2019a, Mathworks Inc., MA, USA) software. The average value of three measurements in fully expanded leaves per plant was used for the SPAD value (SPAD-502; Minolta Corporation, Ltd., Osaka, Japan). For the identical growth parameters, five and eight replicates were used for each light treatment at the end of the seedling stage and at harvest (4 weeks after transplanting), respectively. The number of replicates was the same for both exp.1 and exp. 2.

### Photosynthetic parameters

Photosynthetic properties were measured from a fully expanded leaf and in four replicates at the verification experiment using a portable photosynthetic system (LI-6800, LI-COR) at 2 and 3 WAT. Measurement was conducted with a transparent leaf chamber (LI-6800-12 A, 2 × 3 cm, LI-COR) under the same light conditions (white LED light used during cultivation after transplanting with PPFD 200 µmol m^−2^ s^−1^) as cultivation: 60% RH, 800 µmol mol^−1^ of reference CO_2_, and 700 µmol s^−1^ airflow.

### Statistical analysis

The exp. 1 and exp. 2 were replicated two times. The growth and photosynthetic characteristics of butterhead lettuce plants were statistically analyzed by one-way analysis of variance (ANOVA) using the SAS statistical program (Statistical Analysis System, Version 9.4, SAS Institute, Cary, NC, USA). Significant differences in variances among light treatments were compared with Duncan’s multiple range test. For the photosynthetic characteristics of the two treatments at 2 and 3 WAT in a verification experiment, Student’s t-test was used. Every procedure related to optimization was performed using design of experiment (DOE) software (Design-Ease software v.13, 2020, Stat-Ease Inc., Minneapolis, MN, USA). The similarity between response surfaces was calculated as the RMSE value of 250 points on the response values of the surfaces.

### Supplementary Information

**Additional file 1: Figure S1.** Normalizedspectral distributions of red, green and blue LEDs. **Figure S2.** Response surface methodology results based on single growth index, shoot fresh weight (A) and leaf area (B), and multiple growth index of shoot fresh weight and leaf area with 50% and 50% weights respectively (C), 75% and 25% (D) and 25% and 75% (E). The RGB and growth index mean light treatments during seedling stage and parameters measured at harvest, respectively. Numbers for R, G and B under each plot mean the optimal combination percentages, maximizing each growth index.

## Data Availability

The datasets supporting the conclusions of this article are included within the article or are available from corresponding author on reasonable request.
