# Supplementary material for: Optimizing the photon ratio of red, green, and blue LEDs for lettuce seedlings: a mixture design approach
Source: Plant Methods. 2023 Nov 5;19:121. doi: 10.1186/s13007-023-01098-8 (PMC10625695; doi:10.1186/s13007-023-01098-8)
Supplement: Supplementary file 1 — Additional file 1: Figure S1. Normalizedspectral distributions of red, green and blue LEDs. Figure S2. Response surface methodology results based on single growth index, shoot fresh weight (A) and leaf area (B), and multiple growth index of shoot fresh weight and leaf area with 50% and 50% weights respectively (C), 75% and 25% (D) and 25% and 75% (E). The RGB and growth index mean light treatments during seedling stage and parameters measured at harvest, respectively. Numbers for R, G and B under each plot mean the optimal combination percentages, maximizing each growth index. [file 13007_2023_1098_MOESM1_ESM.docx]

**Additional file 1**


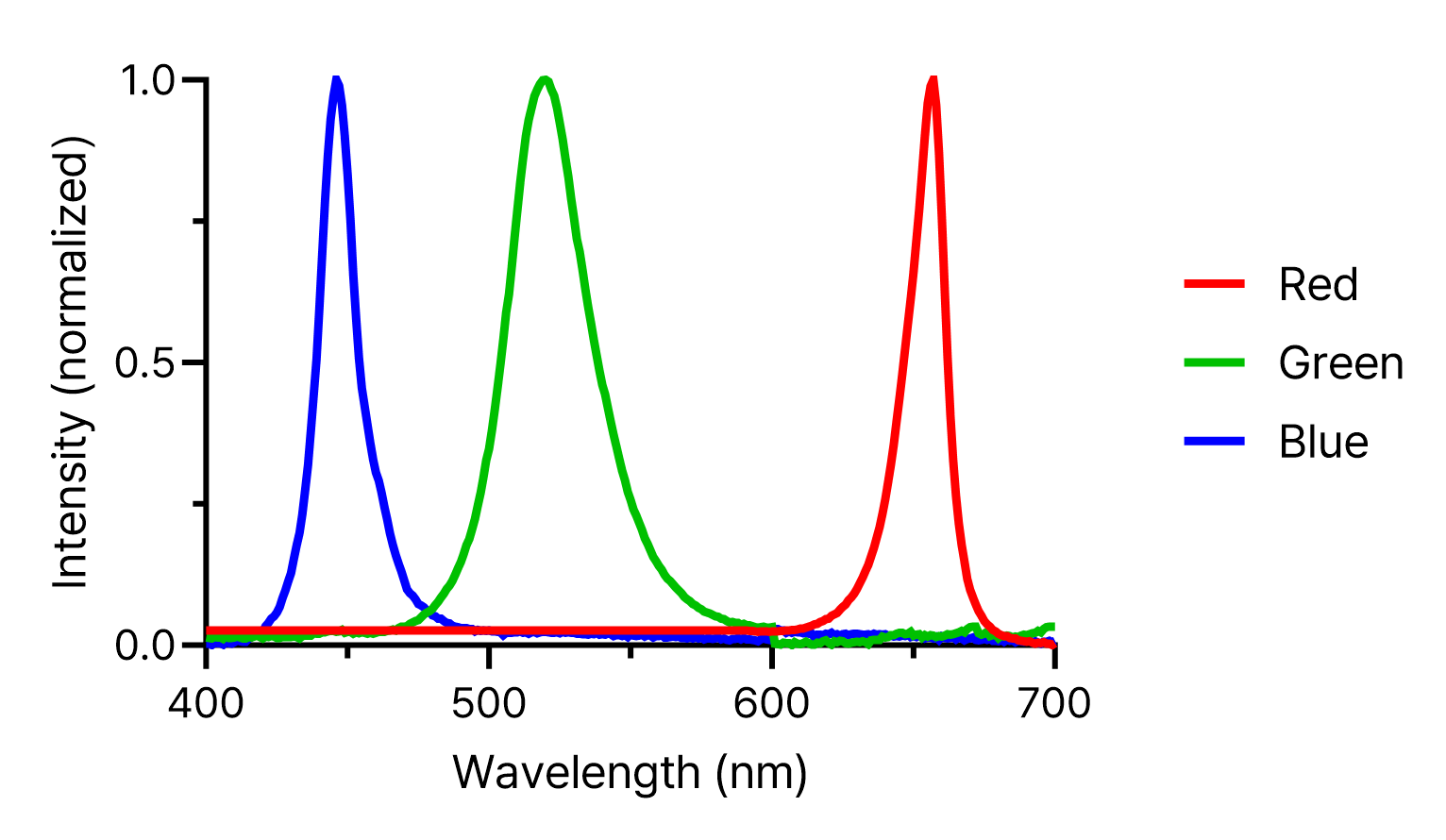


**Fig. S1.** Normalized spectral distributions of red, green and blue LEDs.


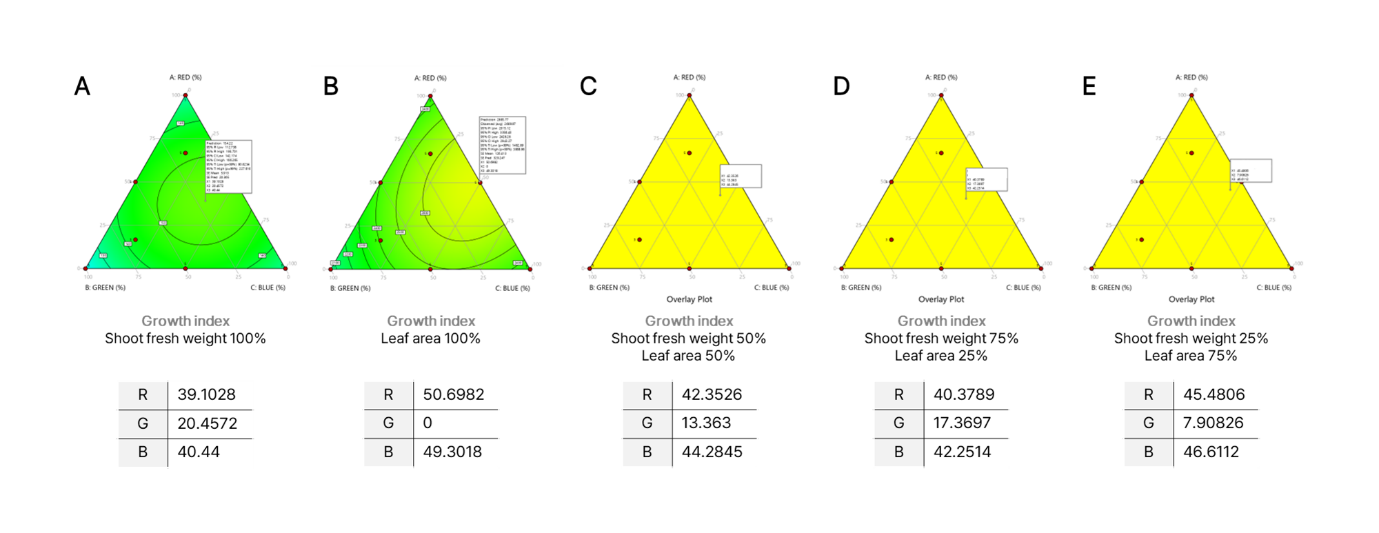


**Fig. S2.** Response surface methodology results based on single growth index, shoot fresh weight (A) and leaf area (B), and multiple growth index of shoot fresh weight and leaf area with 50% and 50% weights respectively (C), 75% and 25% (D) and 25% and 75% (E). The RGB and growth index mean light treatments during seedling stage and parameters measured at harvest, respectively. Numbers for R, G and B under each plot mean the optimal combination percentages, maximizing each growth index.
